# Lockdown effects on Parkinson’s disease during COVID-19 pandemic: a pilot study

**DOI:** 10.1007/s13760-021-01732-z

**Published:** 2021-07-01

**Authors:** Marika Falla, Alessandra Dodich, Costanza Papagno, Alessandro Gober, Pamela Narduzzi, Enrica Pierotti, Markus Falk, Francesca Zappini, Carlo Colosimo, Luca Turella

**Affiliations:** 1 Center for Mind/Brain Sciences-CIMeC, University of Trento, Rovereto, TN Italy; 2 Psychology Department, Università degli studi di Milano‐Bicocca, Milan, Italy; 3 Institute of Mountain Emergency Medicine, Eurac Research, Bolzano, Italy; 4 Department of Neurology, Santa Maria University Hospital, Terni, Italy

**Keywords:** Parkinson’s disease, Telemedicine, Motor symptoms, Non-motor symptoms, COVID-19, Psychological performance

## Abstract

The coronavirus-disease 2019 (COVID-19) outbreak precipitated prolonged lock-down measures. The subsequent social distancing, isolation, and reduction in mobility increased psychological stress, which may worsen Parkinson’s disease (PD). Therefore, telemedicine has been proposed to provide care to PD patients. To evaluate the effects of lock-down on motor and nonmotor symptoms in PD patients during the COVID-19 pandemic and the feasibility of telemedicine. Motor and nonmotor aspects were longitudinally assessed using structured questionnaires at baseline (in-person, February 2020) and at follow-up (remote web-based video, lock-down) evaluation. Of the seventeen PD patients evaluated at baseline, fourteen agreed to participate in, and completed follow-up evaluations. There was an impairment of nonmotor aspects measured with the MDS-UPDRS part I (*p* < 0.001) during lock-down. Nine patients participated independently in the telemedicine evaluation while five needed help from relatives. Our preliminary findings suggest an impairment of nonmotor symptoms in PD patients and support the feasibility and need for telemedicine in monitoring PD patients during the COVID-19 pandemic, to guarantee optimal assistance with reducing the burden of infection. Our findings also suggest that movement disorder clinics should be carefully considering socio-demographics and clinical features when developing telemedicine programs.

**Supplementary Information:**

The online version contains supplementary material available at 10.1007/s13760-021-01732-z.

## Introduction

Parkinson’s disease (PD) is a chronic and progressive neurodegenerative disease affecting 1% of the population above 60 years [1]. The impact of Coronavirus-disease 2019 (COVID-19) pandemic on a vulnerable population, such as PD patients, highlights the practical concerns of clinical management in PD. The COVID-19 outbreak elicited prolonged lock-down measures in Italy from March 9th to May 4th, 2020. The subsequent reduction of mobility for most of the people, along with social distancing and isolation measures, increased psychological stress [2], which may worsen the progression of PD [3]. The use of telemedicine has been proposed for healthcare delivery during the COVID-19 pandemic [4], and the International Parkinson and Movement Disorder Society released a step-by-step guide on implementing telemedicine in a movement disorders clinic [5]. Indeed, the remote and web-based video motor evaluation of PD patients has been reported to be as feasible, valid, and reliable as the in-person assessment [6, 7]. Only two items (rigidity and retropulsion) in the Movement Disorders Society-Unified Parkinson disease rating Scale (MDS-UPDRS) part III require hands-on assessment; the reliability and validity of the modified motor UPDRS, but not MDS-UPDRS excluding the aforementioned items, have been verified [8]. Moreover, the MDS-UPDRS part III motor scores for in-clinic and telemedicine visits have not yet been compared. Due to their advanced age and potentially limited familiarity with web-based tools, PD patients may have difficulty being introduced to clinical practice. In this study, we aimed to obtain data on the effects of lockdown measures on motor and nonmotor symptoms of PD in a small sample of patients currently enrolled in a research project and to evaluate the feasibility of a remote, web-based video evaluation for PD patients during the COVID-19 pandemic.

## Materials and methods

### Study population and assessment

This study is an extension of an ongoing longitudinal “Study of the neural bases underlying the beneficial effects of physical activity in Parkinson's disease”, which was interrupted by the COVID-19 outbreak. The extension study protocol was approved by the Institutional Review Board (Protocol Number 2019-033) of Trento. Participants’ inclusion criteria were diagnosis of idiopathic PD based on the MDS criteria [9] and disease severity ≤ 3 based on the modified Hoehn and Yahr scale (H&Y) [10]. Exclusion criteria were the presence of severe comorbidities, neurological (e.g., DSM-IV-dementia) and psychiatric disorders. The participants gave written informed consent. The baseline evaluation was performed on seventeen patients in February 2020 with a face-to-face interview and hands-on clinical neurological examination at the Center for Neurocognitive Rehabilitation at Trento University, Italy. The follow-up (FU) evaluation was performed using an online standardized platform, according to privacy requirements, during the last week (April 24th–May 1st 2020) of the COVID-19 lock-down period in Italy. Feasibility was measured by the completion of telemedicine evaluation, alone or with the help of relatives. Demographics and clinical information [e.g., age, gender, age at PD onset, disease duration, PD phenotype: bradykinetic/rigid or tremor-dominant, PD side onset, education and web-based tool (PC) use] were collected along with data on PD therapy, expressed as levodopa daily dose equivalent (LEDD) [11]. We administered several structured questionnaires at baseline and at FU evaluations, with the exception of the Montreal Cognitive Assessment (MoCA) for global cognitive status, H&Y (both performed only at baseline) and the Perceived Stress Scale (PSS) (performed only at FU) [12, 13]. Motor and nonmotor symptoms were assessed during the “ON” state, through the revised MDS- UPDRS, which includes part I [non-motor aspects of experiences of daily living (EDL)], part II (motor aspects of EDL), part III (motor evaluation) and part IV (motor complications) [14]. PD severity was assessed with H&Y staging [10]. Psychosocial well-being was assessed with the 12-item Parkinson Anxiety Scale (OR-PAS), the 30-item Geriatric Depression Scale (GDS), the self-administered and informant Apathy Evaluation Scale (AES-S, AES-I), the Lubben Social Network Scale-Revised (LSNS-R), and the 39-item Parkinson’s Disease Questionnaire (PDQ-39) with summary Index (PDQ-39-SI) calculation [15–20]. The balance confidence was assessed using the Falls Efficacy Scale International (FES-I) and the Activity-specific Balance Confidence scale (ABCs-I) [21, 22]. The freezing of gait was recorded using the New Freezing of Gait Questionnaire (NFOGQ) [23] and the Fatigue Severity Scale (FSS) was also administered [24]. A screening for COVID-19 symptoms was carried out; participants were asked if they had experienced the following symptoms since the beginning of the lock-down period: fever, chills, repeated shaking with chills, headache, sore throat, muscle pain, and a new loss of taste or smell [25].

### Statistical analysis

Data are presented as frequencies and percentages for categorical data, and as mean with standard deviation (SD) for continuous data. Since outliers were present, nonparametric methods were used to assess inter- and intra-group differences by means of the Wilcoxon-Signed-Rank Test and the Mann–Whitney-*U*-test. The Chi-Square-test was used for counting data. Due to the large number of variables and the low number of cases, a factor analysis was performed using principal components with varimax rotations on differences from baseline to FU for LEDD, MDS-UPDRS total score, H&Y, AES (S and I), OR-PAS, GDS, PDQ-39, FES-I, FSS, ABCs-I, and NFOGQ. Eigenvalues > 1 were used to identify the number of factors, and factor loadings > 0.7 were used for variable identification since correlation coefficients of approximately 0.7 can be detected with a sample size of 14. Post-hoc group comparisons for variables associated with a factor were performed only for factors demonstrating significant group differences. All *p*-values are two-sided and corrected for multiple comparisons using the Jianjun Li procedure [26]; a value less than 0.05 was considered statistically significant. SPSS software (IBM version 26.00) was used.

## Results

Of the seventeen PD patients evaluated at baseline, fourteen agreed to participate in the telemedicine evaluation. Three patients did not participate: two due to limited knowledge or availability of web-based tools, and one did not respond. All fourteen participants completed the telemedicine evaluation and no unexpected events or technical problems (that could affect the quality of the assessment) were reported. Nine patients participated in the web-based video evaluation independently (independent PC use), while five patients required assistance from relatives. Baseline demographic and clinical data are shown in Table 1.

**Table 1:** Baseline demographic and clinical data of the 14 PD patients

Features/variables	Mean ± SD; (median, min–max) or *N*(%)
Age, years	64.9 ± 8.5; (66.5, 49–77)
Gender, men	7 (50)
Body side PD onset, right	9 (64.3)
Age at PD onset, years	59.2 ± 8.2; (61, 40–71)
PD duration, years	5.7 ± 4.1; (5, 1–17)
PD phenotype, PIGD/TD	3 (21.4)/11 (78.6)
Education, years	12.8 ± 4; (13, 8–18)
PC use, alone/with help	9 (64.3)/5 (42.9)
MoCA (cut off < 17.363)	22.5 ± 1.9; (24.5,19.9–25.3)
[*N* < cut off, %]	1 (7.1)
Job, retired/working	11 (78.6); 3 (21.4)

*MoCA* Montreal Cognitive Assessment; *N* number; *PD* Parkinson disease; *PIGD* postural instability and gait difficulty; *TD* tremor-dominant

Data on motor and psychometric performance at baseline and FU are reported in Table 2. One patient reported an episodic fever without any other symptoms related to COVID-19 disease at FU evaluation. Thirteen out of 14 patients were on PD medications; nine had no PD-therapy modification at FU, while three had PD-therapy modification (two had therapy reductions secondary to increased levodopa-induced dyskinesia and one had increased therapy due to worsening of tremor and bradykinesia). MDS-UPDRS total score increased (*p* = 0.023) between the two evaluations, with the effect mainly driven by part I (*p* < 0.001). Specifically, two patients reported worsening in cognition, one in hallucinations and psychosis (presence phenomena), eight in anxiety, two in apathy, five in sleep problems, eight in daytime sleepiness, three in pain/other sensations, five in urinary problems, five in constipation, three in lightheadedness on standing, and six in fatigue. AES-S and AES-I mean scores were not different in the longitudinal evaluations, however, the number of apathetic patients in caregiver’s report (AES-I) and self-evaluation (AES-S) was different both at baseline (*p* = 0.043) and at follow-up (*p* = 0.009). GDS mean scores were within the normal range on both evaluations, but individual scores were positive for depression at baseline in five patients (and remained positive in four at FU). OR-PAS scores were positive at baseline and increased significantly at FU (*p* = 0.007) due to avoidance behaviour (*p* < 0.001). Persistent anxiety was present both at baseline and follow-up. LSN-S and PDQ39-SI did not change. Mean scores in FES-I were in the range of moderate concern for falling on both evaluations, with a trend towards increased fear of falling (*p* = 0.064). ABCs and FSS mean scores were within normal ranges on both evaluations. Four of the 6 patients with FOG reported worsening and two patients reported FOG only at FU.

**Table 2:** Baseline and follow-up data on motor and psychometric performances of the 14 PD patients

Features/variables	Baseline (February)	Follow-up (end of April)	*p*-value (change over time)
LEDD (mg/day)	551.3 ± 343.7; (468, 0–1220)	471.3 ± 321.2; (410, 0–1220)	0.144
Levodopa	13 (92.9)	13 (92.9)	
Dopamine agonists	9 (64.3)	9 (64.3)	
MAOB-inhibitors	4 (28.6)	4 (28.6)	
COMT-inhibitors	1 (7.1)	1 (7.1)	
MDS-UPDRS score Part I	6.2 ± 2.9; (7, 2–12)	9.8 ± 4.6; (9, 2–17)	**0.001**
MDS-UPDRS score Part II	8.9 ± 4.6; (9.5, 0–16)	10.1 ± 4.7; (9.5, 3–18)	0.375
MDS-UPDRS score Part III	15.5 ± 5.8; (14, 9–30)	16.3 ± 7.4; (14, 8–36)	0.497
MDS-UPDRS score Part IV	2.2 ± 3.3; (0.5, 0–9)	3 ± 4.6; (0, 0–13)	0.900
MDS-UPDRS total score	32.8 ± 10.8; (32.5, 13–54)	39 ± 13.6; (34.5, 19–57)	**0.023**
H&Y score	1.7 ± 0.6; (2, 1–3)	1.7 ± 0.6; (2, 1–3)	1.000
AES-S (cut-off > 37)	29.1 ± 5.1; (30, 21–37)	27.9 ± 5.1; (27.5, 20–37)	0.231
[*N* > cut off, %]	(0)	(0)	
AES-I (cut-off > 37)	34.45 ± 9; (35, 22–48)	36 ± 9.3; (37.5, 17–50)	0.959
[*N* > cut off, %]	(6, 42.9)	(8, 57.1)	
OR-PAS persistent (cut-off > 4.5)	8 ± 5.2; (8.5, 0–14)	7.4 ± 4.4; (8, 0–15)	0.617
[*N* > cut off, %]	(8, 57.1)	(9, 64.3)	
OR-PAS episodic (cut-off > 4.5)	2 ± 2.7; (1, 0–10)	1.9 ± 2.8; (1, 0–9)	0.905
[*N* > cut off, %]	(1, 7.1)	(2, 14.3)	
OR-PAS avoidance behaviour (cut-off > 3.5)	1.8 ± 2.2; (1, 0–7)	7.5 ± 1.7; (8, 3–9)	**0.001**
[*N* > cut off, %]	(3, 21.4)	(13, 92.8)	
OR-PAS total (cut-off > 8.5)	11.8 ± 8.4; (12.5, 3–31)	16.9 ± 7.4; (16.5, 7–32)	**0.007**
[*N* > cut off, %]	8 (57.2)	11 (78, 6)	
GDS (cut-off > 10)	8.1 ± 7.1; (7.5, 0–20)	6.7 ± 5.6; (5.5, 0–18)	1.000
[*N* > cut off, %]	5 (35.7)	4 (28, 6)	
PDQ39-SI	19.8 ± 9.8; (16.5, 7.3–37.2)	15.9 ± 6.8; (16.1, 7- 30.5)	0.221
LSNS-R	36.1 ± 7.7; (35, 19–47)	36.1 ± 9.2; (37.5, 17–50)	1.000
PSS (cut-off > 14)		10.7 ± 4.8; (11, 6–23)	
[*N* > cut off, %]		(2, 14.3)	
FES-I (cut-off > 16)	22.9 ± 7.2; (21, 16–30)	26.9 ± 8.2; (24.5, 17–47)	0.064
[*N* > cut off, %]	(14, 100)	(14, 100)	
FSS (cut-off > 4.5)	3.8 ± 1.9; (4.6, 1–6.1)	3.4 ± 1.9; (3.2, 1.3–6.5)	0.397
[*N* > cut off, %]	(6, 42.9)	(5, 35.7)	
ABCs-I (cut-off < 50%)	79.6 ± 23.1; (90.6, 34.4–98.7)	77.8 ± 18.7; (85.3, 28.1–94.4)	0.345
[*N* > cut off, %]	(4, 28.5)	(3, 21.4)	
NFOGQ	6.8 ± 9.9; (0, 0–26)	5.1 ± 8.3; (0, 0–25)	0.624
[*N* > cut off, %]	(6, 42.9)	(6, 42.9)	
Physical activity (minutes/week)	266.2 ± 103.6; (200, 60–480)	223.6 ± 120.3; (210, 0–480)	0.138

Values expressed as mean ± SD; (median, min–max) or *N*(%). Statistically significant values are reported in bold

*ABCs-I* Activity specific Balance Confidence scale International; *AES-S* Apathy Evaluation Scale-Self; *AES-I* Apathy Evaluation Scale-informant; *FES-I* Falls Efficacy Scale International; *FSS* Fatigue Severity Scale; *GDS* Geriatric Depression Scale; *H&Y* modified Hoehn and Yahr scale; *LEDD* levodopa equivalent daily dose; *LSNS–R* Lubben Social Network scale revised; *MDS-UPDRS* Movement Disorder Society-Unified Parkinson’s Disease Rating Scale; *N* number; *NFOGQ* new freezing of gait questionnaire; *PAS* Parkinson Anxiety Scale; *PDQ-39* Parkinson’s Disease Questionnaire 39-Items

Factor analysis identified three factors, and the respective factor loadings for parameter differences from baseline to FU are provided in Table 3. The factor “Balance” positively correlated with FES and negatively with ABCs. The factor “Parkinson” was positively correlated with MDS-UPDRS and negatively with NFOGQ. The factor “Psychosocial well-being” positively correlated with OR-PAS, GDS, and PDQ39. These three factors were further evaluated in relation to several patient characteristics (age, age at PD onset, PC-use, PD-side, PD-type, education, PD-duration, and gender) (Table 3). The analysis showed that the PC-use dependent patients had a significant improvement at FU in PDQ39 (*p* = 0.05) and GDS (*p* = 0.05) and trended towards improvement in OR-PAS (*p* = 0.07) compared to their PC-use independent counterparts (See Figures S1 in the Supplementary Material for comprehensive image analysis). Left PD-side onset patients demonstrated a trend towards improvement on the ABCs-I (*p* = 0.08) compared to their right PD-side onset counterparts (Fig. S1).

**Table 3:** Rotated component matrix^a^

Variable	Balance	Parkinson	Psycho-social well-being	PC use	PD side		Age	Age at onset	PD type	Education (years)	PD duration	Gender
	Factor loadings			*p*-values (Mann–Whitney-*U* test)								
Δ FES	**0.93**	− 0.17	0.14	0.25	0.59	1.00		0.44	0.18	0.38	0.48	0.75
Δ ABCs-I	** − 0.89**	− 0.10	0.01	0.50	**0.08**	0.90		0.25	0.02	0.89	0.30	0.41
Δ MDS-UPDRS	0.01	**0.82**	− 0.10	0.59	0.12	0.95		0.65	0.88	0.23	0.12	0.37
Δ PDQ39-SI	0.19	− 0.04	**0.81**	**0.05**	0.55	0.23		0.41	0.39	0.55	0.16	0.85
Δ GDS	0.48	− 0.35	**0.70**	**0.05**	0.41	0.84		0.55	0.33	0.41	0.69	0.51
Δ OR-PAS	− 0.17	0.11	**0.88**	**0.07**	0.64	0.07		0.65	0.81	0.38	0.70	0.65
Δ FSS	0.57	0.69	0.09	0.95	0.64	0.09		0.14	0.48	0.32	0.70	0.41
Δ NFOGQ	0.19	** − 0.71**	0.04	0.53	0.17	0.89		0.55	0.47	0.68	0.11	0.14
Δ AES	− 0.45	0.56	− 0.16	0.13	0.27	0.32		0.32	0.07	0.33	0.84	0.14
Balance				0.39	0.64	0.48		0.23	0.24	0.55	0.70	0.41
Parkinson				0.39	**0.10**	0.34		0.57	0.31	0.10	0.25	0.57
Psychosocial well-being				**0.03**	0.95	0.14		0.57	0.48	0.74	0.44	0.95

Extraction Method: Principal Component Analysis. Rotation Method: Varimax with Kaiser Normalization

Mann–Whitney *U* and Wilcoxon *W* for PC use, PD side, age, age at onset, PD type, education, PD duration, gender data

Difference from baseline and follow-up Δ: *ABCs-I* Activity specific Balance Confidence scale International; *AES* Apathy Evaluation Scale; *FES-I* Falls Efficacy Scale International; *FSS* Fatigue Severity Scale; *GDS* Geriatric Depression Scale; *MDS-UPDRS* Movement Disorder Society-Unified Parkinson’s Disease Rating Scale; *NFOGQ* new freezing of gait questionnaire; *PAS* Parkinson Anxiety Scale; *PDQ-39* Parkinson’s Disease Questionnaire 39-Items

## Discussion

The preliminary data show an impairment of nonmotor symptoms in PD patients during the lockdown. There was an increase in MDS-UPDRS total score in the prospective evaluation, particularly due to most of the nonmotor aspects of experiences of daily living (e.g., cognition, hallucinations, and psychosis, anxiety, apathy, sleep problems, daytime sleepiness pain/other sensations, urinary problems, constipation, lightheadedness on standing and fatigue). A similar study recently reported a worsening in non-motor symptoms (e.g., anxiety and cognition) [27].

A worsening of anxiety was reported by eight patients in part I of MDS-UPDRS but was not observed in the OR-PAS evaluation. An increase in OR-PAS at FU was related exclusively to avoidance behaviours imposed by the lock-down measures. Consistent with the literature, anxiety (OR-PAS) was present in nearly 60% of PD patients [28, 29]. A worsening in apathy was reported by two patients in part I of MDS-UPD but was not observed in the AES-S where none of the patients reported apathy (AES-S), which is in disagreement with the caregiver’s report (AES-I) and previous work. This is likely due to the small sample size in our study and different patient characteristics across the studies (age, PD duration and stage, cognition, apathy scale employed) [30–34]. Consistent with previous work, depression (GDS) was present in 35% of PD patients [35]. Longitudinal evaluations of apathy (AES-S and AES-I) and depression (GDS) did not demonstrate significant differences during lock-down. Motor performance only demonstrated a trend towards increased fear of falling as measured by FES-I. UPDRS part III was not different at follow-up and this may be due to the maintenance of physical activity (Table 2) during the lockdown in our sample.

Fasano and colleagues reported no difference in COVID-19 risk and mortality in nonadvanced PD patients compared to the general population [36], whereas in advanced PD patients with COVID-19 has been reported a high mortality rate ranging from 40–50% [37] to 20% [38, 39], particularly in those with lengthy disease duration, the use of advanced therapies and particularly in the older population [37, 40]. Artusi and colleagues [41] recently reviewed all the published articles reporting data on PD with a confirmed diagnosis of COVID-19 showing a prevalence below 1% except for two studies of patients living in Lombardy, Italy (prevalence of 7 and 8.5%) [36, 42]. They also found an overall hospitalization rate of 28.6% and mortality rate of 18.9% in PD patients [41].

Until a definitive control of COVID-19 disease is achieved, reduced social interaction and use of face masks may remain major indications particularly for moderate to severe PD patients. Despite the limited number of patients in our study, 82% of our patients successfully completed telemedicine evaluation, supporting its feasibility during the COVID-19 pandemic. The data further suggest that the help of relatives may be crucial for PD patients with limited knowledge or availability of web-based tools. Households may overall be beneficial for the subgroup of patients dependent on PC use, especially during the lock-down. Indeed, such patients reported a significant improvement in quality of life (PDQ-39) and depression (GDS), with a trend towards improvement in anxiety (PAS) compared with those independent of PC use. Telemedicine evaluation appears to help reduce the burden of infection in PD patients. Our preliminary results suggest that socio-demographics (e.g., availability of web-based tools, relative support) and clinical (e.g., disease severity) features of PD patients should be carefully considered by movement disorder clinics when developing telemedicine programs [43].

The impairment in the nonmotor aspects observed in our small cohort of PD patients during the lock-down supports the need for telemedicine in PD patient care in agreement with the International Parkinson and Movement Disorder Society guides [5]. The preliminary data show that it was feasible to monitor the most mild-to-moderate PD patients with telemedicine evaluation during the COVID-19 pandemic, but that the lack of relatives and limited knowledge or availability of web-based tools may present potential limitations for widespread applicability in Italy.

This study has limitations: main limitations are the limited number of patients and the use of face-to-face questionnaires during the first evaluation and of a remote evaluation in the follow-up evaluation. Although the reliability and validity of the modified motor UPDRS without rigidity and pull test are proven for remotely administration [8], MDS-UPDRS, which is derived from UPDRS, has not yet been validated remotely and/or used for comparison between in-clinic and telemedicine visit scores. Non-motor and self-rating scales can likely be administered face-to-face and via telemedicine with similar results, but the telemedicine evaluation has also not yet been tele-validated. Further we acknowledge the absence of a time-matched control group due to the unusual circumstance where the entire world was in lockdown. It would be interesting to expand the results collecting further data post-lockdown.

Our preliminary results support the feasibility of tele-evaluation in PD patients and encourage studies in larger samples to validate the scale application in such modalities, in agreement with Schneider et al. [44]. Moreover, the current findings should be investigated on a larger sample, and possibly with a control group, to gauge whether the nonmotor worsening observed was due to heightened psychological stress from the lock-down period or if it was related to other causes (e.g., the natural progressive course of the disease).

## Conclusions

Our findings suggest an impairment of non-motor symptoms in PD patients during COVID-19 lockdown. Our preliminary data also support the feasibility and need for telemedicine in monitoring PD patients, during the COVID-19 pandemic, to guarantee optimal assistance with reducing the burden of infection. Our findings also suggest that movement disorder clinics should be carefully considering socio-demographics and clinical features when developing telemedicine programs.

## Supplementary Information

Below is the link to the electronic supplementary material.

Supplementary file1 (DOCX 112 KB)

## Data Availability

Data available on request.

## References

[1] Tysnes OB, Storstein A (2017). Epidemiology of Parkinson's disease. J Neural Transm (Vienna).

[2] Venkatesh A, Edirappuli S (2020). Social distancing in covid-19: what are the mental health implications?. BMJ.

[3] Helmich RC, Bloem BR (2020). The impact of the COVID-19 pandemic on Parkinson’s disease: hidden sorrows and emerging opportunities. J Parkinson Dis.

[4] Hollander JE, Carr BG (2020). Virtually perfect? Telemedicine for COVID-19. N Engl J Med.

[5] International Parkinson and Movement Disorder Society. https://www.movementdisorders.org/MDS/About/Committees--Other-Groups/Telemedicine-in-Your-Movement-Disorders-Practice-A-Step-by-Step-Guide.htm. Accessed 9 April 2021

[6] Dorsey ER, Deuel LM, Voss TS, Finnigan K, George BP, Eason S (2010). Increasing access to specialty care: a pilot, randomized controlled trial of telemedicine for Parkinson's disease. Mov Disord.

[7] Biglan KM, Voss TS, Deuel LM, Miller D, Eason S, Fagnano M (2009). Telemedicine for the care of nursing home residents with Parkinson's disease. Mov Disord.

[8] Abdolahi A, Scoglio N, Killoran A, Dorsey ER, Biglan KM (2013). Potential reliability and validity of a modified version of the unified Parkinson's disease rating scale that could be administered remotely. Parkinsonism Relat Disord.

[9] Postuma RB, Berg D, Stern M, Poewe W, Olanow CW, Oertel W (2015). MDS clinical diagnostic criteria for Parkinson's disease. Mov Disord.

[10] Hoehn MM, Yahr MD (1967). Parkinsonism: onset, progression and mortality. Neurology.

[11] Tomlinson CL, Stowe R, Patel S, Rick C, Gray R, Clarke CE (2010). Systematic review of levodopa dose equivalency reporting in Parkinson's disease. Mov Disord.

[12] Nasreddine ZS, Phillips NA, Bédirian V, Charbonneau S, Whitehead V, Collin I (2005). The montreal cognitive assessment, MoCA: a brief screening tool for mild cognitive impairment. J Am Geriatr Soc.

[13] Cohen S, Kamarck T, Mermelstein R (1983). A global measure of perceived stress. J Health Soc Behav.

[14] Goetz CG, Tilley BC, Shaftman SR, Stebbins GT, Fahn S, Martinez-Martin P (2008). Movement disorder society UPDRS revision task force. Movement disorder society-sponsored revision of the unified Parkinson's Disease Rating Scale (MDS-UPDRS): scale presentation and clinimetric testing results. Mov Disord.

[15] Santangelo G, Falco F, D'Iorio A, Cuoco S, Raimo S, Amboni M (2016). Anxiety in early Parkinson's disease: validation of the Italian observer-rated version of the Parkinson Anxiety Scale (OR-PAS). J Neurol Sci.

[16] Yesavage JA, Brink TL, Rose TL, Lum D, Huang V, Adey M et al (1982-1983) Development and validation of a geriatric depression screening scale: a preliminary report. J Psychiatr Res 17:37–49 10.1016/0022-3956(82)90033-4 7183759

[17] Marin RS, Biedrzycki RC, Firinciogullari S (1991). Reliability and validity of the apathy evaluation scale. Psychiatry Res.

[18] Lubben J (1998). Assessing social networks among elderly populations. Fam Commun Health J Health Promot Maintenance.

[19] Jenkinson C, Fitzpatrick R, Peto V, Greenhall R, Hyman N (1997). The Parkinson’s Disease Questionnaire (PDQ-39): development and validation of a Parkinson’s disease summary index score. Age Ageing.

[20] Peto V, Jenkinson C, Fitzpatrick R, Greenhall R (1995). The development and validation of a short measure of functioning and well being for individuals with Parkinson’s disease. Qual Life Res.

[21] Yardley L, Beyer N, Hauer K, Kempen G, Piot-Ziegler C, Todd C (2005). Development and initial validation of the falls efficacy scale-international (FES-I). Age Ageing.

[22] Powell LE, Myers AM (1995). The activities-specific balance confidence (ABC) scale. J Gerontol Med Sci.

[23] Nieuwboer A, Rochester L, Herman T, Vandenberghe W, Emil GE, Thomaes T (2009). Reliability of the new freezing of gait questionnaire: agreement between patients with Parkinson’s disease and their careers. Gait Posture.

[24] Krupp LB, LaRocca NG, Muir-Nash J, Steinberg AD (1989). The fatigue severity scale. Application to patients with multiple sclerosis and systemic lupus erythematosus. Arch Neurol.

[25] Centers for Disease Control and Prevention. https://www.cdc.gov/coronavirus/2019-ncov/symptoms-testing/symptoms.html. Accessed 9 April 2021

[26] Jianjun L (2007). A two-step rejection procedure for testing multiple hypotheses. J Stat Plan Infer.

[27] Palermo G, Tommasini L, Baldacci F, Del Prete E, Siciliano G, Ceravolo R (2020). Impact of coronavirus disease 2019 pandemic on cognition in Parkinson's disease. Mov Disord.

[28] Lin CH, Lin JW, Liu YC, Chang CH, Wu RM (2015). Risk of Parkinson’s disease following anxiety disorders: a nationwide population-based cohort study. Eur J Neurol.

[29] Chaudhuri KR, Schapira AH (2009). Non-motor symptoms of Parkinson’s disease: dopaminergic pathophysiology and treatment. Lancet Neurol.

[30] Marin RS, Fogel BS, Hawkins J, Duffy J, Krupp B (1995). Apathy: a treatable syndrome. J Neuropsychiatry Clin Neurosci.

[31] McKinlay A, Grace RC, Dalrymple-Alford JC, Anderson TJ, Fink J, Roger D (2008). Neuropsychiatric problems in Parkinson’s disease: comparisons between self and caregiver report. Aging Ment Health.

[32] Radakovic R, Davenport R, Starr JM, Abrahams S (2018). Apathy dimensions in Parkinson’s disease. Int J Geriatr Psychiatry.

[33] Schiehser DM, Liu L, Lessig SL, Song DD, Obtera KM, Burke Iii MM (2013). Predictors of discrepancies in Parkinson’s disease patient and caregiver ratings of apathy, disinhibition, and executive dysfunction before and after diagnosis. J Int Neuropsychol Soc.

[34] Valentino V, Iavarone A, Amboni M, Moschiano F, Picillo M, Petretta V (2018). Apathy in Parkinson's disease: differences between caregiver's report and self-evaluation. Funct Neurol.

[35] Reijnders JS, Ehrt U, Weber WE, Aarsland D, Leentjens AF (2008). A systematic review of prevalence studies of depression in Parkinson’s disease. Mov Disord.

[36] Fasano A, Cereda E, Barichella M, Cassani E, Ferri V, Zecchinelli AL, Pezzoli G (2020). COVID-19 in Parkinson's disease patients living in Lombardy, Italy. Mov Disord.

[37] Antonini A, Leta V, Teo J, Chaudhuri KR (2020). Outcome of Parkinson's disease patients affected by COVID-19. Mov Disord.

[38] Fasano A, Elia AE, Dallocchio C, Canesi M, Alimonti D, Sorbera C, Alonso-Canovas A, Pezzoli G (2020). Predictors of COVID-19 outcome in Parkinson's disease. Parkinsonism Relat Disord.

[39] Zhang Q, Schultz JL, Aldridge GM, Simmering JE, Narayanan NS (2020). Coronavirus disease 2019 case fatality and Parkinson's disease. Mov Disord.

[40] Papa SM, Brundin P, Fung VSC, Kang UJ, Burn DJ, Colosimo C, Chiang HL, Alcalay RN, Trenkwalder C, MDS-Scientific Issues Committee (2020). Impact of the COVID-19 pandemic on Parkinson's disease and movement disorders. Mov Disord.

[41] Artusi CA, Romagnolo A, Ledda C, Zibetti M, Rizzone MG, Montanaro E, Bozzali M, Lopiano L (2021). COVID-19 and Parkinson's disease: what do we know so far?. J Parkinsons Dis.

[42] Cilia R, Bonvegna S, Straccia G, Andreasi NG, Elia AE, Romito LM, Devigili G, Cereda E, Eleopra R (2020). Effects of COVID-19 on Parkinson's disease clinical features: a community-based case-control study. Mov Disord.

[43] Motolese F, Magliozzi A, Puttini F, Rossi M, Capone F, Karlinski K, Stark-Inbar A, Yekutieli Z, Di Lazzaro V, Marano M (2020). Parkinson's disease remote patient monitoring during the COVID-19 lockdown. Front Neurol.

[44] Schneider RB, Myers TL, Tarolli CG, Amodeo K, Adams JL, Jensen-Roberts S (2020). Remote administration of the MDS-UPDRS in the time of COVID-19 and beyond. J Parkinsons Dis.

