# Supplementary material for: Lockdown effects on Parkinson’s disease during COVID-19 pandemic: a pilot study
Source: Acta Neurol Belg. 2021 Jul 1;121(5):1191–8. doi: 10.1007/s13760-021-01732-z (PMC8248756; doi:10.1007/s13760-021-01732-z)
Supplement: Supplementary file 1 — Supplementary file1 (DOCX 112 KB) [file 13760_2021_1732_MOESM1_ESM.docx]

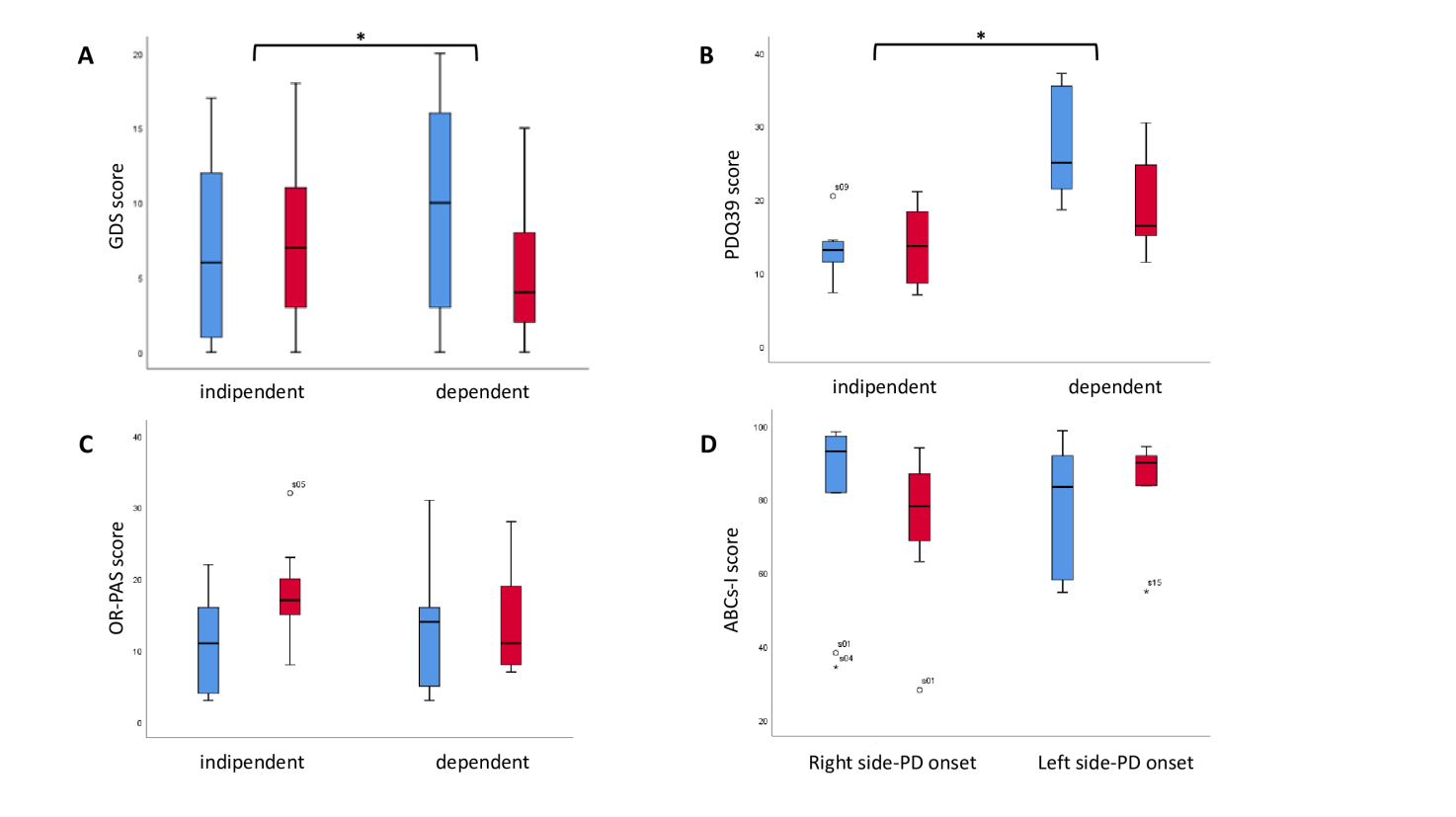


FIG. S1. Post-hoc group comparison for variables (PC-use and PD- side) significantly associated with the factors (Balance, Parkinson, Psychosocial well-being). Bars represent mean values and standard deviation. Blue represents baseline and red follow-up evaluations. (A) GDS score in PD patients PC-use independent and dependent (B) PDQ-39 score in PD patients PC-use independent and dependent (C) OR-PAS score in PD patients PC-use independent and dependent (D) ABCs-I score in PD patients with left PD-side and right PD-side onset.
